# Betaine promotes osteogenic differentiation in immortalized human dental pulp-derived cells

**DOI:** 10.1038/s41405-022-00123-7

**Published:** 2022-10-07

**Authors:** Chatvadee Kornsuthisopon, Dusit Nantanapiboon, Sunisa Rochanavibhata, Nunthawan Nowwarote, Worachat Namangkalakul, Thanaphum Osathanon

**Affiliations:** 1 Dental Stem Cell Biology Research Unit, Faculty of Dentistry, Chulalongkorn University, Bangkok, Thailand; 2 Dental Material Research and Development Center and Department of Operative Dentistry, Faculty of Dentistry, Chulalongkorn University, Bangkok, Thailand; 3 Department of Oral and Maxillofacial Surgery, Faculty of Dentistry, Chulalongkorn University, Bangkok, Thailand; 4 Centre de Recherche des Cordeliers, Universite Paris Cite, Sorbonne Universite, INSERM UMRS 1138, Molecular Oral Pathophysiology, Paris, France; 5 Department of Oral Biology, Faculty of Dentistry, Universite Paris Cite, Paris, France; 6 Department of Anatomy, Faculty of Dentistry, Chulalongkorn University, Bangkok, Thailand

**Keywords:** Dental pulp, Endodontics

## Abstract

**Objectives:**

This study aimed to evaluate the effect of betaine (BET) on immortalized human dental pulp stem cell (ihDP) osteogenic differentiation.

**Materials and methods:**

hDPs were immortalized using SV40 T-antigen transfection. Characterization, multilineage differentiation, proliferation, cell cycle, colony-forming unit, and cellular senescence were evaluated (*n* = 4). The effect of BET on ihDP response was assessed (*n* = 4). Osteogenic differentiation was detected using ALP, ARS staining, and RT-qPCR (*n* = 4). To investigate the involvement of calcium signaling, the cells were pretreated with either 8-(NN-diethylamino)octyl-3,4,5-trimethoxybenzoate (TMB-8) or thapsigargin before BET treatment (*n* = 6).

**Results:**

ihDPs retained similar phenotypic characteristics presented in hDPs but exhibited an increase in cell proliferation and extended culture to passage 25. An increased proportion of cells in S and G2/M phases without senescence was observed in ihDPs. BET (50 mM) treatment significantly increased mineral deposition at 14 days and upregulated *ALP*, *MSX2*, *BMP2*, and *RUNX2* expression. TMB-8 pretreatment reduced the effect of BET-induced ihDP osteogenic differentiation, whereas thapsigargin promoted osteogenic differentiation in ihDPs synergistically with BET.

**Conclusion:**

ihDPs showed superior proliferation ability and a longer life span, which could serve as a promising cell for regenerative dentistry. BET promoted odonto/osteogenic differentiation via intracellular calcium regulation.

## Introduction

Dental pulp is a vascularized connective tissue that occupies the pulpal cavity of the tooth. It provides various essential functions, including the formation of reparative dentin to protect the dental pulp tissues from the external environment and the maintenance of tooth homeostasis as a viable organ [1]. The ability of dental pulp to differentiate toward odontoblasts following dental pulp injury suggests its potential regenerative ability [2–4]. Moreover, the dental pulp stem cells possess self-renewal and multilineage differentiation properties toward several lineages such as osteogenic, chondrogenic, adipogenic, neurogenic, odontogenic, and myogenic lineages [5]. Therefore, this dental stem cell could be a valuable source for prospecting translational research; however, several limitations are the major obstacles to stem cell therapy [6].

Betaine (BET) is a trimethylglycine derivative, which controls various biological processes such as inflammation, apoptosis, and osteoblast differentiation [7–9]. BET treatment enhanced osteogenic differentiation of human adipose-derived stem cells [10] and human osteoblast cells [11] demonstrated by upregulation of alkaline phosphatase activity, mineral deposition, and expression of osteogenic marker gene expressions, such as *RUNX2*, *OSX*, *OCN*, *OPN*, and *BSP*. Moreover, BET induced the calcium influx from extracellular components through the L-type calcium channel and calcium/calmodulin-dependent kinase II, leading to the activation of extracellular-signal-regulated kinase (ERK) signaling, one of the key signaling pathways involved in osteoblastogenesis. BET treatment additionally increased IGF-I production, which enhances osteoblast differentiation and bone formation [11]. Therefore, BET could be a promising bioactive compound for promoting mineralized tissue regeneration. The present study aimed to investigate the effect of BET on odonto/osteogenic differentiation of immortalized human dental pulp stem cells (ihDPs).

## Materials and methods

### Primary cell culture of hDPs

The protocol for cell isolation was approved by the Human Research Ethics Committee, Faculty of Dentistry, Chulalongkorn University (No. 017/2022). Cells were isolated from the impacted third molars that required surgical removal according to the patient’s treatment plan. The dental pulp tissues were collected, minced, and cultured on 35-mm tissue culture dishes. The cells were cultured in a growth medium consisting of Dulbecco’s Modified Eagle Medium (Cat. no. 11960, Gibco, USA) supplemented with 10% fetal bovine serum (Cat. no. 10270, Gibco, USA), 100 unit/mL penicillin, 100 μg/mL streptomycin, 250 ng/mL amphotericin B (Antibiotic–Antimycotic, cat. no. 15240, Gibco, USA), and 1% L-glutamine (GlutaMAX-1, cat. no. 35050, Gibco, USA) and incubated in 5% CO_2_ humidified atmosphere at 37 °C. The cells in passage 3 were subjected to immortalization.

### Immortalization of hDPs

hDPs were transfected with pSV3neo (ATCC no. 37150), a plasmid containing coding sequences of SV40 T-Ag and neomycin (G418)-resistance gene, as described previously [12, 13]. Transfection was performed using SuperFect Transfection Reagent (Qiagen, Germany) in those cells (8 × 10^4^ cells/well) in the 24-well plates. Cell selection was performed by maintaining the transfected cells in a growth medium supplemented with 100 µg/mL G418 (Thermo Fisher Scientific, USA).

In some experiments, cells were treated with 50 mM BET (Cat. no. 107437, Sigma-Aldrich, USA). To investigate the influence of BET on calcium influx, cells were pretreated with 10 µM thapsigargin (Cat. no. 1138/1, R&D Systems, USA) or 50 µM TMB-8 (Cat. no. 53464725, Sigma-Aldrich, USA) before being stimulated with 50 mM BET (*n* = 6).

### Stem cell characterization

The hDPs and ihDPs were analyzed for mesenchymal stem cell (MSC) surface protein expression using a flow cytometry (*n* = 4). Single-cell suspensions were stained with fluorescence-conjugated antibodies at a 1:50 dilution as follows: FITC conjugated anti-human CD44 (Cat. No. 555478, BD Bioscience, USA), PE-conjugated anti-human CD105 (Cat. No. 21271054, Immuno Tools, Germany), FITC-conjugated anti-human CD90 (Cat. No. ab124527, Abcam, USA), and FITC-conjugated anti-CD45 (Cat. No. 21810455, Abcam, USA) antibodies. Mean fluorescence intensity was determined and analyzed using FACS^Calibur^ flow cytometer and CellQuest software (BD Bioscience, USA). Multilineage differentiation toward adipogenic and osteogenic lineages was also performed (*n* = 4).

### Differentiation assay

For osteogenic differentiation, the cells (2.5 × 10^4^ cells/well in 24-well plates) were cultured in an osteogenic medium containing a growth medium supplemented with 250 nM dexamethasone (Cat. no. D8893, Sigma-Aldrich, USA), 50 µg/mL ascorbic acid (Cat. no. A-4034, Sigma-Aldrich, USA), and 5 mM β-glycerophosphate (Cat. no. G9422, Sigma-Aldrich, USA) for 14 days. Alkaline phosphatase enzymatic activity (ALP) and mineralization were examined using ALP staining and alizarin red S (ARS) staining, respectively (*n* = 4).

For the adipogenic differentiation, the cells (1.25 × 10^4^ cells/well) were maintained in 24-well plates with adipogenic medium comprising a growth medium containing 1 µM dexamethasone (Cat. no. D8893, Sigma-Aldrich, USA), 0.1 mg/mL insulin (Cat. no. 11070738 Sigma-Aldrich, USA), 0.2 mM indomethacin (Cat. no. 53861, Sigma-Aldrich, USA), and 1 mM IBMX (Cat. no. PHZ1124, Thermo Fisher Scientific, USA) for 16 days. The intracellular lipid droplet accumulation was examined using oil red o staining (*n* = 4).

### Alkaline phosphatase staining

The cells (*n* = 4) were fixed with 4% paraformaldehyde solution for 10 min, followed by incubating with BCIP/NBT tablets (Roche, USA) for 30 min in a dark environment at room temperature. The ALP-stained cells were observed under the inverted microscope (Olympus, USA).

### Mineralization assay

The cells (*n* = 4) were fixed with cold methanol for 10 min and subsequently rinsed with deionized water. The cells were stained ARS staining (Sigma-Aldrich, USA) for 3 min at room temperature. The excess stain was washed twice with deionized water. The staining was observed under the inverted microscope (Olympus, USA). For semi-quantitative analysis, stained deposits were solubilized using 10% cetylpyridinium chloride monohydrate (Sigma-Aldrich, USA) in 10 mM sodium phosphate. The absorbance was measured at 570 nm with a microplate reader (Biotek ELX800, USA).

### Oil red O staining

The cells (*n* = 4) were fixed with 10% buffered formalin for 30 min, followed by incubating with 0.2% Oil red O solution for 15 min. Lipid accumulation was examined using an inverted microscope (Olympus, USA).

### Cell viability and proliferation

The cells (1.25 × 10^4^ cells/well) were seeded in 24-well plates (*n* = 4). At designated time points, the cells were incubated with a 0.5 mg/mL MTT solution for 15 min at 37 °C to allow formazan crystal formation. The precipitated crystals were solubilized in a dimethyl sulfoxide and glycine buffer. The absorbance at 570 nm was measured using a microplate reader (Biotek ELX800, USA) and the percentage cell number was calculated and normalized with the control.

### Cell cycle analysis

The cells (1 × 10^5^ cells/well) were seeded in 12-well plates (*n* = 4). On day 3, cells were trypsinized and fixed with 70% ethanol at −20 °C for 30 min. Ribonucleic acid (RNA) elimination was performed by adding 2 µL of 4 mg/mL RNase A (Cat. no. EN0531, Thermo Fisher Scientific, USA). The cells were stained with 4 µg/mL propidium iodide and analyzed by a FACS^Calibur^ flow cytometer using CellQuest software (BD Bioscience, USA).

### Colony-forming unit assay

The cells (500 cells/well) were seeded in 6-well plates and maintained in a normal growth medium for 14 days (*n* = 4). The cells were fixed with 4% buffered formalin for 10 min and stained with coomassie blue (Sigma-Aldrich, USA). The colony-forming unit was investigated using an inverted microscope (Olympus, USA). The stained cells were eluted with 5% (v/v) methanol and 7.5% (v/v) acetic acid solution. The absorbance was read at 667.5 nm using a microplate reader (Biotek ELX800, USA).

### Senescence assay

The cells (2.5 × 10^4^ cells/well) were seeded in 24-well plates and cultured for 3 days (*n* = 4). The cells were fixed with 4% paraformaldehyde for 8 min at room temperature. Subsequently, the cells were stained with senescence‐associated β‐galactosidase chromogenic substrate solution (Sigma-Aldrich, USA) at 37 °C for 16 h. The β‐galactosidase-positive staining was observed using an inverted microscope (Olympus, USA).

### Reverse transcription polymerase chain reaction (RT-qPCR)

Total cellular RNA was extracted using TRIzol reagent (RiboEx solution, cat. no. 301-001, GeneAll, South Korea) and subsequently converted to complementary DNA using ImProm-II Reverse Transcription System (Cat. no. A3800, Promega, USA) (*n* = 6). RT-qPCR was performed using the CFX Connect Real-Time PCR machine (Bio-Rad, Singapore) with FastStart Essential DNA Green Master (Roche Diagnostic, Germany). The amplification profile was: 95 °C/20 s, 60 °C/20 s, and 72 °C/20 s for 45 cycles. Melt curve analysis was performed to determine product specificity. The mRNA levels of the target genes were normalized to the expression of *GAPDH*. The relative gene expression was quantified by the 2^−ΔΔCt^ method [14]. The oligonucleotide sequences used are presented in Supplementary Table 1.

### Statistical analyses

All experiments were performed at least in quadruplicate. The statistical analysis was performed using Prism 8 (GraphPad Software, USA). The data were presented as mean ± standard deviation. Each dot represented the individual values. The Mann–Whitney *U* test was used for two independent group comparisons. For three or more group comparisons, statistical differences were assessed using the Kruskal–Wallis test followed by a pairwise comparison. Statistical significance was considered when *P* < 0.05.

## Results

### Characterization of immortalized human dental pulp stem cells (ihDPs)

**Fig. 1:**
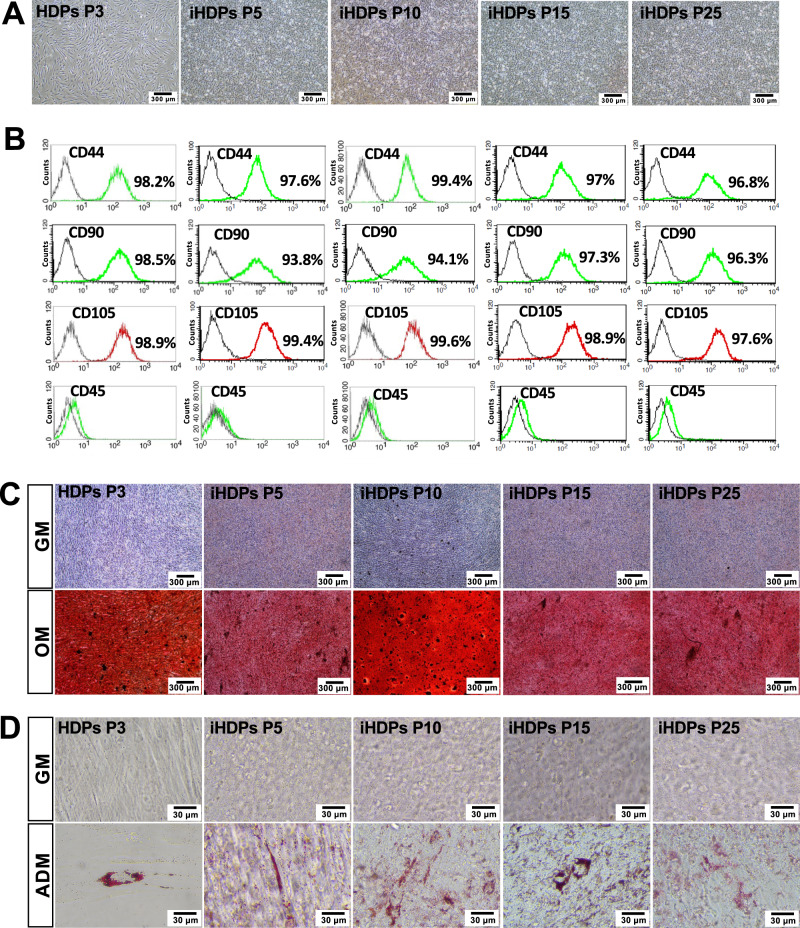
Characterization of ihDPs. Morphological observation of hDPs (passage 3) and ihDPs (passages 5, 10, 15, 25, and 50) using phase-contrast microscopy. Scale bars: 300 µm (**A**). Evaluation of stem cell surface markers using flow cytometry (**B**). Multilineage differentiation potential toward osteogenic lineage. Scale bars: 300 µm (**C**). Oil red O staining after adipogenic induction. Scale bars: 30 µm (**D**).

hDPs (passage 3) had a spindle-shaped and fibroblast-like morphology. ihDPs (passages 5, 10, 15, and 25) showed similar spindle-like morphology, although their shapes were not as distinctive as hDPs (Fig. 1A). Flow cytometry analysis revealed that both hDPs and ihDPs showed comparable expressions of MSC-related surface markers (CD44, CD90, and CD105) and the absence of the hematopoietic cell marker CD45 (Fig. 1B). Both hDPs and ihDPs exhibited a significant increase in mineral deposition after osteogenic induction compared to undifferentiated control cells (*P* = 0.0286) (Fig. 1C). Furthermore, the intracellular lipid accumulation was observed in both hDPs and ihDPs maintaining in an adipogenic induction medium as compared with those cultured in a growth medium (Fig. 1D). Thus, ihDPs retained the characteristics of MSCs and their multipotency ability.

### Immortalized human dental pulp stem cell proliferation

**Fig. 2:**
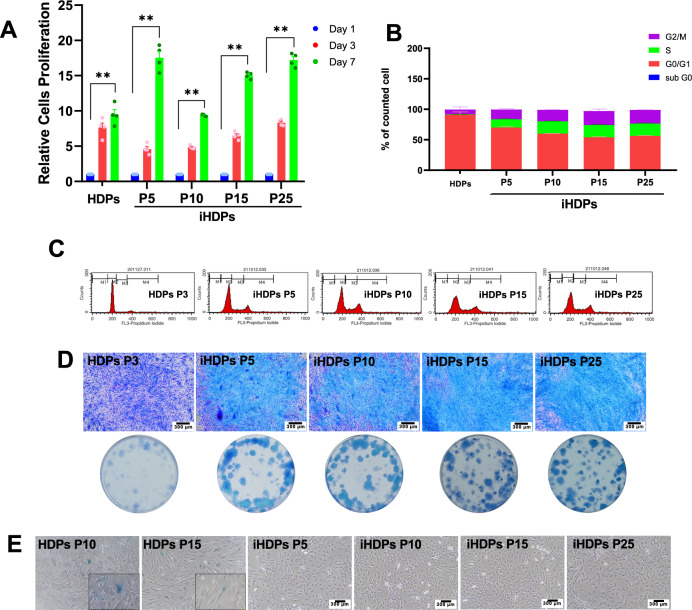
Cell proliferation of ihDPs. Cell proliferation was evaluated using an MTT assay on days 1, 3, and 7 (**A**). Cell cycle analysis was performed using flow cytometry (**B**, **C**). Colony-forming unit assay. Scale bars: 300 µm (**D**). Senescence-associated β-galactosidase activity. Scale bars: 300 µm (**E**). The inset images showed the β-galactosidase-positive cells. Bars indicate a significant difference between groups (*P < 0.05, **P < 0.01).

Cell proliferation of ihDPs was determined by the cell viability assay, colony-forming unit ability, and cell cycle analysis. Cell viability was assessed using the MTT assay on days 1, 3, and 7. The proliferation of hDPs (passage 3) and ihDPs (passages 5, 10, 15, and 25) was gradually increased (*P* = 0.1448 for hDPs and *P* = 0.2205 for ihDPs at day 3), with a significant upregulation on day 7 (*P* = 0.0055 for hDPs and *P* = 0.0028 for ihDPs) (Fig. 2A). The cell cycle assay revealed that hDPs exhibited a higher percentage of counted cells in the G0/G1 phase, while ihDPs showed a higher number of cells in the S and G2-M phases as compared with hDPs (Fig. 2B, C). Accordingly, increased colony-forming size and density were significantly observed in ihDPs (Fig. 2D). The senescent cells were found in hDPs as shown by β-galactosidase activity (passages 10 and 15), but no cellular senescence was observed in all passages of ihDPs (Fig. 2E).

### BET promotes ihDP osteogenic differentiation

**Fig. 3:**
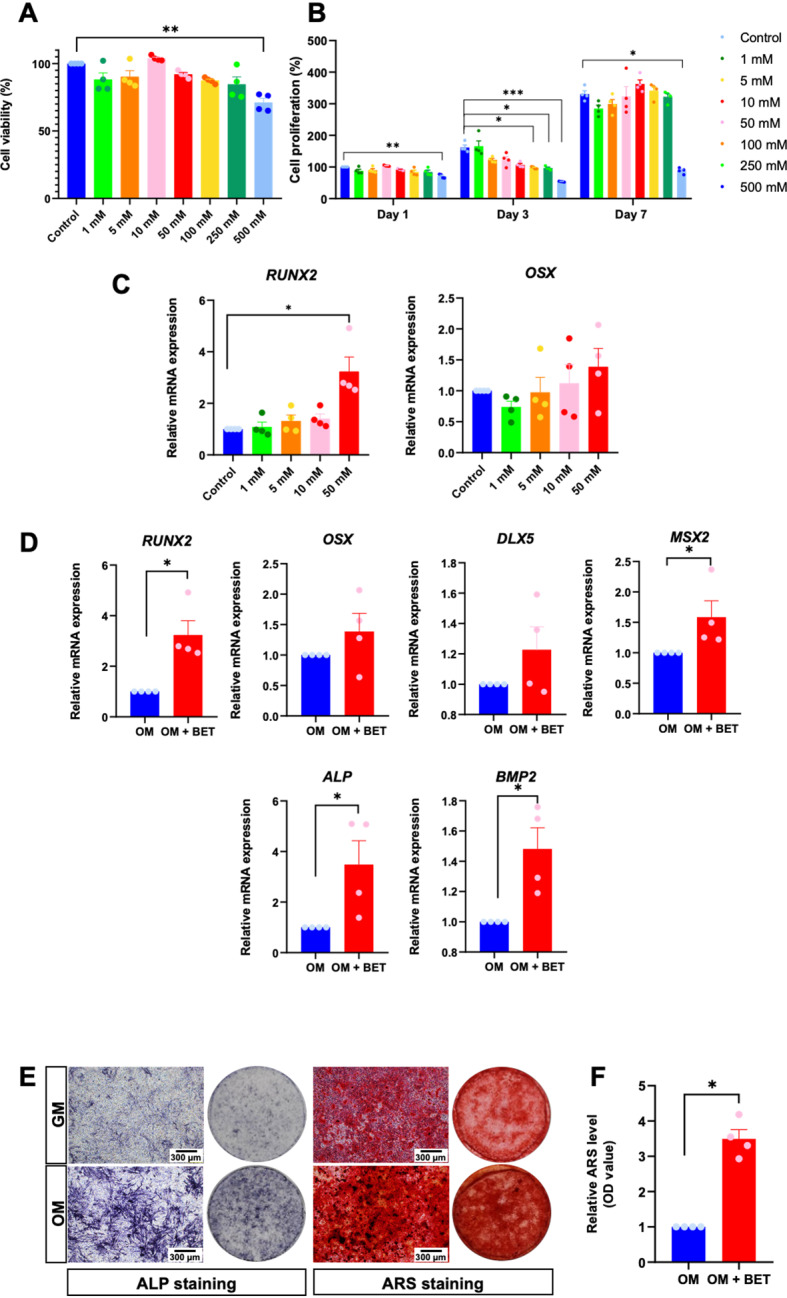
BET promotes osteogenic differentiation in ihDPs. Cell viability and proliferation were evaluated using an MTT assay (**A**, **B**). ihDPs were treated with 1–50 mM BET and maintained in an osteogenic induction medium for 3 h. mRNA expression of osteogenic-related genes was evaluated using real-time polymerase chain reaction (**C**). Representative ALP and ARS red S staining images of ihDPs after 50 mM BET treatment for 7 and 14 days. Scale bars: 300 µm (**D**). Quantification of ARS red S staining was shown (**E**). Bars indicate a significant difference between groups (*P < 0.05, **P < 0.01, ***P < 0.001).

BET was previously reported to enhance osteogenic differentiation in several types of cells [10, 11]. To evaluate the optimal concentration of BET, cell viability and proliferation assay were examined. ihDP cells were maintained in a growth medium supplemented with 1, 5, 10, 50, 100, 250, and 500 mM BET. Only 500 mM BET was toxic to the cells after 24 h of treatment (*P* = 0.0071) (Fig. 3A). Cell proliferation was assessed on days 1, 3, and 7 by the MTT assay. A significant decrease in cell proliferation was found in 100, 250, and 500 mM BET treatment on day 3 (*P* = 0.0226, *P* = 0.0213, *P* = 0.0005, respectively) (Fig. 3B). Cell proliferation was significantly attenuated when treating ihDPs with 500 mM BET for 7 days (*P* = 0.0295) (Fig. 3B). Furthermore, the cells were cultured in an osteogenic induction medium supplemented with 1, 5, 10, and 50 mM for 3 h. 50 mM BET-treated ihDPs exhibited a significant upregulation of *RUNX2* mRNA expression (*P* = 0.0169) while *OSX* expression was slightly increased (Fig. 3C). Therefore, 50 mM BET was chosen for subsequent experiments with ihDPs.

To further evaluate the influence of BET on ihDP osteogenic differentiation, the cells were cultured in an osteogenic induction medium supplemented with 50 mM BET. The significant upregulation of osteogenic-related genes, *RUNX2, MSX2*, *ALP*, and *BMP2*, was found after 50 mM BET exposure for 3 h (*P* = 0.0286) (Fig. 3D). Indeed, the increase in ALP and ARS staining was detected after 7 and 14 days of culture with the 50 mM BET treatment, respectively (Fig. 3E). A significant upregulation of relative ARS level was noted in the BET-treated group (*P* = 0.0286) (Fig. 3F).

### Influence of BET on calcium influx

**Fig. 4:**
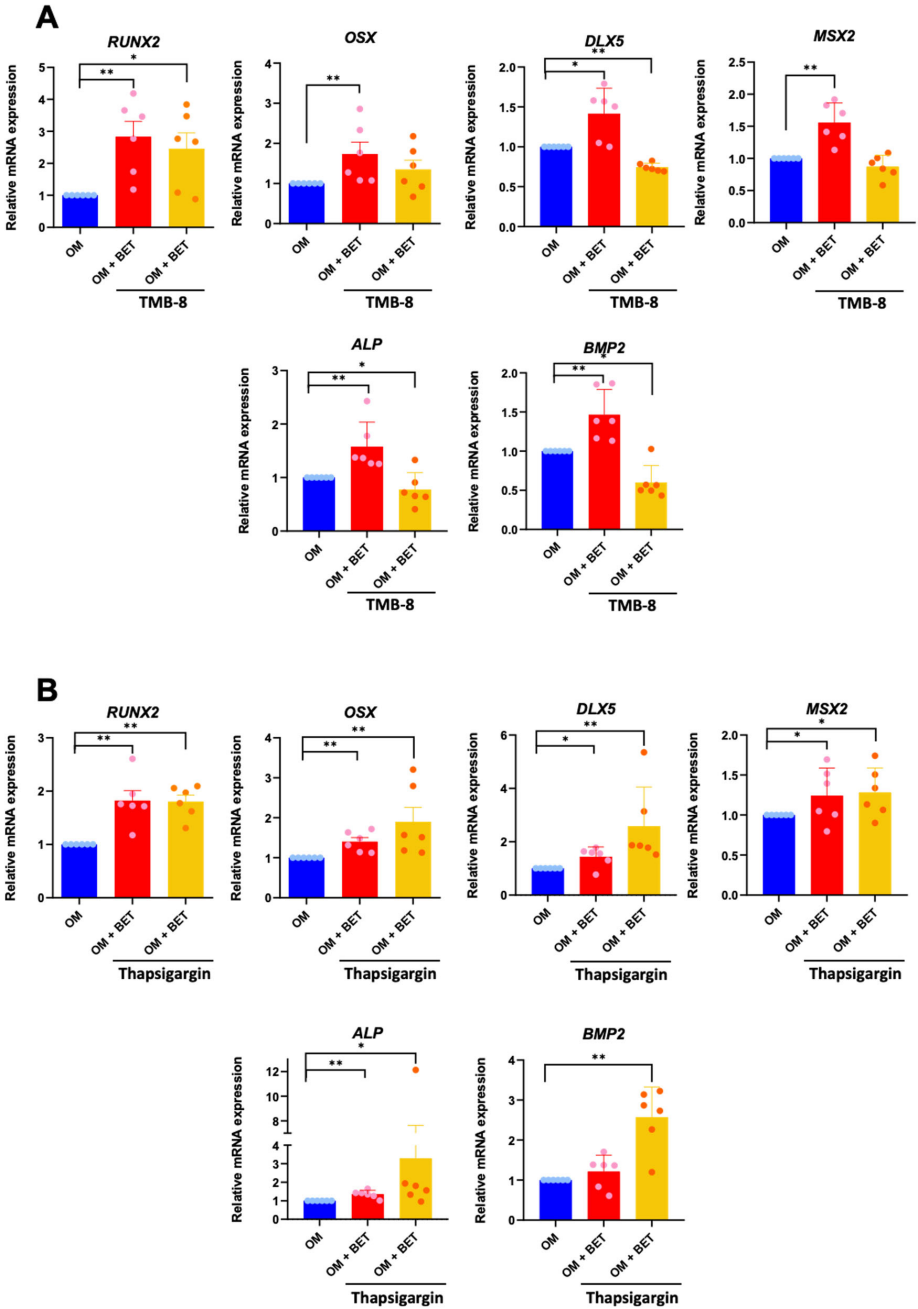
Influence of BET on calcium influx. ihDPs were pretreated with either 50 µM TMB-8 or 10 µM thapsigargin for 30 min prior to stimulating with 50 mM BET for 3 h. The mRNA expression of osteogenic-related genes was examined using a real-time polymerase chain reaction (**A**, **B**). Bars indicate a significant difference between groups (*P < 0.05, **P < 0.01).

A previous study reported that BET promoted osteogenic differentiation of human osteoblast-like cells by inducing a calcium influx from the extracellular milieu [11]. Intracellular calcium antagonist (TMB-8) and agonist (thapsigargin) were used to study whether the effect of BET-induced ihDP osteogenic differentiation was associated with the increased intracellular calcium via calcium influx. ihDPs were pretreated with either 50 µM TMB-8 or 10 µM thapsigargin for 30 min. Subsequently, the cells were stimulated with 50 mM BET in an osteogenic medium for 3 h. The results demonstrated that 50 mM BET treatment significantly upregulated *RUNX2, OSX, DLX5, MSX2*, *ALP*, and *BMP2* mRNA expression (*P* = 0.0022, *P* = 0.0022, *P* = 0.0152, *P* = 0.0022, *P* = 0.0022, *P* = 0.0022, respectively) (Fig. 4A). TMB-8 attenuated the effect of BET-induced ihDP osteogenic differentiation, as demonstrated by significant downregulation of *DLX5*, *ALP*, and *BMP2* mRNA expression after TMB-8 pretreatment (*P* = 0.0476, *P* = 0.0022, *P* = 0.0476, *P* = 0.0476, respectively) (Fig. 4A). For thapsigargin experiment, a similar activity of BET on in vitro osteogenic differentiation of ihDPs was observed. Increased expression of *RUNX2*, *OSX*, *DLX5*, *MSX2*, and *ALP* mRNA expression was found after 50 mM BET treatment (*P* = 0.0022, *P* = 0.0022, *P* = 0.0476, *P* = 0.0476, *P* = 0.0022, respectively) (Fig. 4B). Significant upregulation of *OSX*, *DLX5, MSX2*, *ALP*, and *BMP2* mRNA expression was observed after Thapsigargin pretreatment (*P* = 0.0022, *P* = 0.0022, *P* = 0.0476, *P* = 0.0476, *P* = 0.0022, respectively) (Fig. 4B). However, a comparable mRNA expression level of *RUNX2* was found in the BET treatment and thapsigargin pretreatment group (Fig. 4B).

## Discussion

Dental pulp stem cells are one of the promising cell sources for regenerative dentistry. These cells serve as an in vitro model that helps advance scientific research. However, cell senescence accumulated with ageing limits the growth potential and alters the phenotype as well as biological functions [15]. Here, we investigated the characteristics of ihDPs and evaluated the effect of BET on ihDP osteogenic differentiation potential. ihDPs showed similar characteristics as primary hDPs. However, immortalization influenced cell behavior regarding cell proliferation, cell cycle progression, and cell senescence. BET treatment promoted osteogenic differentiation of ihDPs by inducing intracellular calcium influx.

In the present study, hDPs were immortalized by SV40 T-antigen transfection and the cell behavior was studied. The previous reports demonstrated the effectiveness of SV40 transfection in inducing immortalization of human dental-related cells, including dental pulp stem cells [13, 16–19], periodontal ligament stem cells [15, 20, 21], and cementoblasts [15]. ihDPs were characterized as MSCs according to the guidelines of the International Society for Cellular Therapy [22]. ihDPs exhibited similar cell characteristics regarding cell morphology, MSC-related surface markers, and osteogenic/adipogenic differentiation ability, as observed in hDPs. These findings indicate that ihDPs retain many of the phenotypic characteristics observed in the primary stem cells.

Cell proliferation is a fundamental process in the pulp-dentin tissue healing response [23]. Following the pulp exposure, localized pulp tissue destruction occurs [24]. A cascade of dental pulp stem cell activation and proliferation is required to substitute for those necrotic odontoblasts [25]. The process of cell proliferation is controlled by obligated steps of the cell cycle, which involve cell growth, DNA replication, chromosomal separation, and cell division [26]. In the present study, ihDPs exhibited an extended life span up to passage 25, with superior proliferation as indicated in cell proliferation and colony-forming unit ability. In normal fibroblast cells, telomeres are gradually shortened with each cycle of cell division. When telomeres become critically short, cells basically undergo a senescence [27].

BET was previously reported to enhance osteogenic differentiation in human adipose-derived stem cells [10] and human osteoblast cells [11]. To select the optimal dose for ihDPs, cell viability and proliferation were evaluated after BET treatment. The data indicated that 500 mM was toxic and delayed ihDP proliferation. In addition, 100 and 250 mM BET-treated ihDPs showed a significant decrease in cell proliferation at day 3, thereby only four concentrations of BET (1, 5, 10, and 50 mM) were selected to further examine the effect on in vitro osteogenic differentiation. Due to the superior effect on osteogenic differentiation by inducing a significant upregulation of *RUNX2*, 50 mM BET was chosen for the subsequent experiments. The results showed that BET significantly upregulated osteogenic-related genes (*RUNX2*, *MSX2*, *ALP*, and *BMP2*), ALP activity, and mineralization of ihDPs. Previous studies observed that BET activated the ERK pathway in the osteoblasts [28, 29]. ERK pathway is one of the important signaling cascades that control proliferation and differentiation toward an osteogenic lineage [30]. ERK positively regulates RUNX2, which is the major transcription factor for osteoblast commitment by controlling the expression of several genes essential for osteoblast differentiation [31, 32]. Therefore, we speculated that ERK pathway is one of the possible mechanisms of BET-promoted ihDP osteogenic differentiation. Further investigation is indeed required.

BET-stimulated cytosolic calcium influx was another mechanism of BET-induced osteogenic differentiation that has been reported in human osteoblasts [11]. To evaluate whether BET increased intracellular calcium levels, ihDPs were pretreated with either TMB-8 or thapsigargin prior to BET stimulation. TMB-8 is an intracellular Ca^2+^ antagonist through the inhibition of calcium influx [33], whereas thapsigargin inhibits the endoplasmic calcium pump, which increases cytosolic calcium levels by releasing calcium from intracellular calcium storage [34]. Our results revealed that TMB-8 pretreatment attenuated the effect of BET-induced mRNA expression of osteogenic-related genes. In contrast, pretreatment with thapsigargin showed a synergistic effect with BET, as a higher mRNA expression level of all osteogenic-related genes was observed. These results indicated that BET induced ihDP osteogenic differentiation through mediating intracellular calcium levels.

In conclusion, the present study demonstrated that while retaining many of the phenotypic characteristics observed in primary cells, ihDPs exhibited superior abilities in proliferation with a prolonged life span as compared with hDPs. These biological changes are useful for further application in pulp tissue engineering. In addition, BET is a good candidate to be developed as an adjuvant molecule that promotes osteogenic differentiation of ihDPs. To our best knowledge, this study would be the first investigation that used ihDPs as in vitro models for studying the effects of BET on osteogenic differentiation. However, a limiting factor in their application is that an in vitro experiment performed using ihDPs might not represent all the scenarios that will biologically happen in vivo. Thus, ihDPs would provide basic knowledge regarding the cellular response, which indeed requires further in vivo investigation.

## Supplementary information

Supplementary Table 1
