# Supplementary material for: Betaine promotes osteogenic differentiation in immortalized human dental pulp-derived cells
Source: BDJ Open. 2022 Oct 7;8:31. doi: 10.1038/s41405-022-00123-7 (PMC9546879; doi:10.1038/s41405-022-00123-7)
Supplement: Supplementary file 1 — Supplementary Table 1 [file 41405_2022_123_MOESM1_ESM.docx]

**Betaine promotes osteogenic differentiation in immortalized human dental pulp-derived cells**

Chatvadee Kornsuthisopon^1^, Dusit Nantanapiboon^2^, Sunisa Rochanavibhata^3^, Nunthawan Nowwarote^4,5^, Worachat Namangkalakul^1,6^, Thanaphum Osathanon^1,6^

^1^Dental Stem Cell Biology Research Unit, Faculty of Dentistry, Chulalongkorn University, Bangkok, Thailand

^2^Dental Material Research and Development Center and Department of Operative Dentistry, Faculty of Dentistry, Chulalongkorn University, Bangkok, Thailand

^3^Department of Oral and Maxillofacial Surgery, Faculty of Dentistry, Chulalongkorn University, Bangkok, Thailand

^4^ Centre de Recherche des Cordeliers, Universite Paris Cite, Sorbonne Universite, INSERM UMRS 1138, Molecular Oral Pathophysiology, Paris, France

^5^Department of Oral Biology, Faculty of Dentistry, Universite Paris Cite, Paris, France

^6^Department of Anatomy, Faculty of Dentistry, Chulalongkorn University, Bangkok, Thailand

**Corresponding author**

Thanaphum Osathanon, DDS, Ph.D.

Dental Stem Cell Biology Research Unit, Faculty of Dentistry,

Chulalongkorn University, Bangkok THAILAND 10330

Email: [thanaphum.o@chula.ac.th](mailto:thanaphum.o@chula.ac.th)

**Supplementary Table 1** The oligonucleotide sequences

| Genes | Forward sequences | Reverse sequences | GenBank Accession Number | Size (bp) |
| --- | --- | --- | --- | --- |
| *GAPDH* | 5’-TCATGGGTGTGAACCATGAGAA-3’ | 5’-GGCATGGACTGTGGTCATGAG-3’ | NM_002046.3 | 146 |
| *ALP* | 5’-CGAGATACAAGCACTCCCACTTC-3’ | 5’-CTGTTCAGCTCGTACTGCATGTC-3’ | NM_000478.3 | 120 |
| *BMP2* | 5’-GCGTGAAAAGAGAGACTGC-3’ | 5’-CCATTGAAAGAGCGTCCAC-3’ | NM_001200.4 | 126 |
| *DLX5* | 5’-CAGCCATGTCTGCTTAGACCA-3’ | 5’-ATTCCTGAGACGGATGGTGC-3’ | NM_005221.6 | 197 |
| *MSX2* | 5’-TTACCACATCCCAGCTCCTC-3’ | 5’-CCTGGGTCTCTGTGAGGTTC-3’ | NM_001363626.2 | 111 |
| *OSX* | 5’-GCCAGAAGCTGTGAAACCTC-3’ | 5’-GCTGCAAGCTCTCCATAACC-3’ | NM_001300837.2 | 161 |
| *RUNX2* | 5’-ATGATGACACTGCCACCTCTGA-3’ | 5’-GGCTGGATAGTGCATTCGTG-3’ | NM_001024630.3 | 167 |
